# Novel workflow analysis of robot-assisted hysterectomy through objective performance indicators: a pilot study

**DOI:** 10.3389/fmed.2024.1382609

**Published:** 2024-08-16

**Authors:** Felix Neis, Sara Yvonne Brucker, Armin Bauer, Mallory Shields, Lilia Purvis, Xi Liu, Marzieh Ershad, Christina Barbara Walter, Tjeerd Dijkstra, Christl Reisenauer, Bernhard Kraemer

**Affiliations:** ^1^Department of Obstetrics and Gynecology, University Hospital Tübingen, Tübingen, Germany; ^2^Intuitive Surgical Inc., Sunnyvale, CA, United States

**Keywords:** robot-assisted total hysterectomy, surgical data science, objective performance indicators, surgical workflow, intuitive data recorder, surgical annotation, Da Vinci surgical system

## Abstract

**Introduction:**

The curriculum for a da Vinci surgeon in gynecology requires special training before a surgeon performs their first independent case, but standardized, objective assessments of a trainee’s workflow or skills learned during clinical cases are lacking. This pilot study presents a methodology to evaluate intraoperative surgeon behavior in hysterectomy cases through standardized surgical step segmentation paired with objective performance indicators (OPIs) calculated directly from robotic data streams. This method can provide individual case analysis in a truly objective capacity.

**Materials and methods:**

Surgical data from six robot-assisted total laparoscopic hysterectomies (rTLH) performed by two experienced surgeons was collected prospectively using an Intuitive Data Recorder. Each rTLH video was annotated and segmented into specific, functional surgical steps based on the recorded video. Once annotated, OPIs were compared through workflow analysis and across surgeons during two critical surgical steps: colpotomy and vaginal cuff closure.

**Results:**

Through visualization of the individual steps over time, we observe workflow consistencies and variabilities across individual surgeons of a similar experience level at the same hospital, creating unique surgeon behavior signatures across each surgical case. OPI differences across surgeons were observed for both the colpotomy and vaginal cuff closure steps, specifically reflecting camera movement, energy usage and clutching behaviors. Comparing colpotomy and vaginal cuff closure time needed for the step and the events of energy use were significantly different (*p* < 0.001). For the comparison between the two surgeons only the event count for camera movement during colpotomy showed significant differences (*p* = 0.03).

**Conclusion:**

This pilot study presents a novel methodology to analyze and compare individual rTLH procedures with truly objective measurements. Through collection of robotic data streams and standardized segmentation, OPI measurements for specific rTLH surgery steps can be reliably calculated and compared to those of other surgeons. This provides opportunity for critical standardization to the gynecology field, which can be integrated into individualized training plans in the future. However, more studies are needed to establish context surrounding these metrics in gynecology.

## Introduction

Since the introduction of the da Vinci Surgical System in the 1990s, its use has gained importance in gynecology and other surgical fields (1–3). Since then, Intuitive has established curricula to be completed before the surgeon uses the da Vinci Surgical System in the operating room, as robotic surgery performance has been shown to be dependent on the expertise of the surgeon (4). However, there is still limited understanding of a true surgical learning curve (5). Most studies define the learning curve of robotic surgery via reduced surgical time, docking time, console time, rates of complications, blood drop, and time of hospital stay (5–7). Moreover, many assessments are time-and resource-consuming, subjective, not reproducible, and poorly comparable across surgeons.

One of many ways to look at surgical behavior and the improvement of robotic surgical skills may be the analysis of workflows using individual application of camera movement, energy use and clutching to reposition the hands within the console during specific surgical steps. These robotic data streams can be captured directly from the da Vinci Surgical System using an Intuitive Data Recorder (IDR, Intuitive Surgical Ltd., Sunnyvale, California, United States) (8). Recently, the field of surgical data science has emerged, with a growing interest in objective performance indicators (OPIs), metrics calculated directly from the robotic system’s data streams, that provide truly objective measurements and behaviors within individual surgeries (9, 10). OPIs have been utilized in other surgical specialties to correlate with surgical skill, workflow, and outcomes (11, 12), but no studies have been performed for gynecology procedures.

This feasibility study, which is the first in the gynecology space, introduces a methodology specific robot-assisted total laparoscopic hysterectomy (rTLH) and utilizes it to evaluate surgical workflow and intraoperative behaviors using OPIs. With more research in this space, such objective parameters will enable surgeon proficiency identification and tailored training to the learning surgeon.

## Materials and methods

### Raw data collection and annotation

Six rTLH were performed at the Department of Women’s Health at the University Hospital of Tübingen using the da Vinci Si surgical system (Intuitive Surgical Ltd., Sunnyvale, California, United States). Synchronized video and accompanying robotic data streams were captured using an IDR on loan from Intuitive Surgical for the purpose of this study. All data were encrypted (AES-256) and stored on the Intuitive Data Recorder. Data were copied to an external hard disk (with encrypted access) and sent to Intuitive Surgical for professional annotation. No sound was recorded and neither patient nor surgeon were identifiable from the recordings. Date and time markers were part of the data captured from the da Vinci Surgical System, data allowing identification of the patient was not forwarded to Intuitive. Video annotation started with the insertion of the camera to the abdominal cavity. Video scrubbing algorithm was used to block out endoscope events outside of the body to guarantee patient and OR staff anonymity.

**Figure 1:**
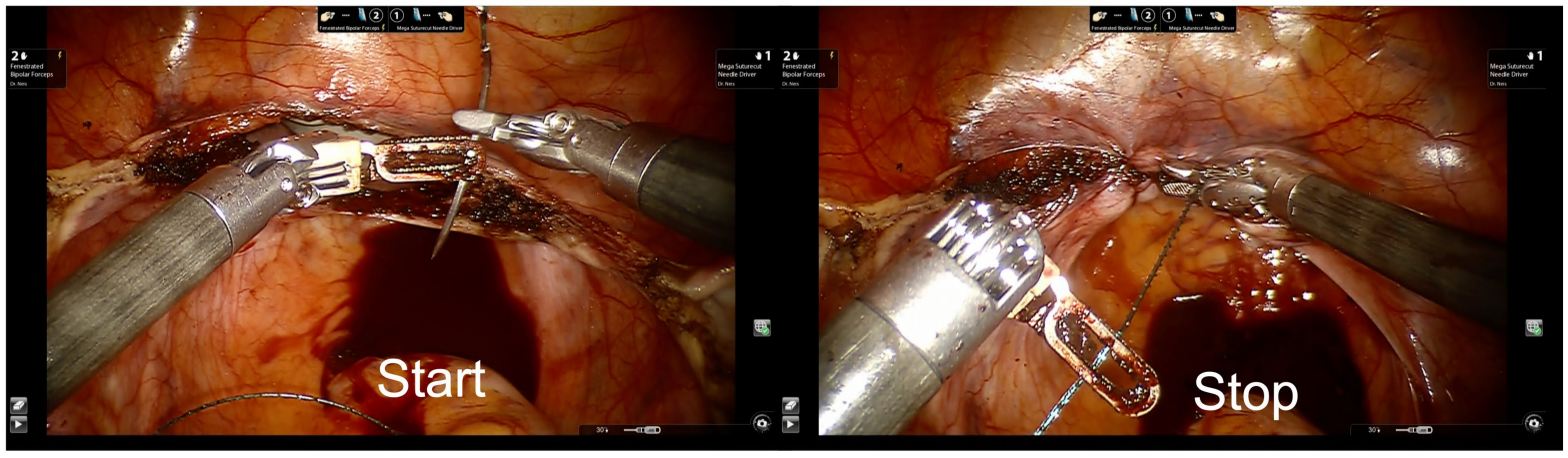
Images depicting start and stop moments for segmentation of hysterectomy. Vaginal cuff closure is presented as an example here. The surgical step begins with the clamping of the needle in the needle holder and ends with the cutting of the barbed thread after complete closure of the colpotomy.

Each video was segmented into functional surgical steps specific to rTLH by a professional annotator (LP), a data scientist trained in gynecologic anatomy and all surgical steps of rTHL. The annotator indicated the start and stop times of each step according to a standardized annotation card (Table 1), which provides a detailed start and stop action for the specific surgical step (Figure 1). Each step may occur multiple times, identifying when a surgeon alternates between steps. As annotations were limited to functional steps, gaps between steps could also be identified. These include cleaning of the camera, change of instruments, surgeon idle time, etc. When a surgeon switched from one surgical step to the next within 2 seconds, no gap would be inserted by the annotator. Metric data that can be extracted via the IDR from the robot are: frequency of camera movement, energy use and clutch use for each side. Within each surgical step these parameters were used to calculate surgical activity within the time parameters of each step. Hereby OPIs can now be analyzed for each individual surgical step. These provide a truly objective measure of surgical behavior that can be attributed to specific rTLH steps. Since during colpotomy and closure of the vaginal cuff camera movement, use of energy and clutch use are frequently applied in every hysterectomy and therefore a large amount of data was available, these two surgical steps were examined in detail.

**Table 1:** Functional surgical steps utilized for annotation during rTLH.

	Surgical step name
1	Mobilize Colon/Removal of Adhesions (optional)
2	Dissection of Fallopian Tube (Left/Right side) (optional)
3	Dissection of IP Ligament (Left/Right side) (optional)
4	Dissection of Utero-Ovarian Ligament (Left/Right side) (optional)
5	Division of the Round Ligament (Left/Right side)
6	Division of the Broad Ligament (Left/Right side)
7	Bladder Flap Creation
8	Division of Uterine Vessels (Left/Right side)
9	Colpotomy
10	Removal of the Uterus
11	Vaginal Cuff Closure

Steps are listed in the order in which they first appear during a standardized laparoscopic hysterectomy. As not all steps might be required during hysterectomy step 1 to 4 are optional steps. The order of the individual steps may vary depending on the individual operation.

### Ethics statement

Ethical approval was obtained from the Ethics Committee of Tübingen University Medical Faculty and University Hospital (621/2018BO1). Three operations, each performed by two experienced da Vinci surgeons, were selected to evaluate the feasibility of the method in this pilot study.

### Statistical analysis

Statistical analysis was performed with Microsoft excel and R version 4.3 and RStudio version 2023.06.1 + 524 using the tidyverse (2.0.0) packages. Statistical comparisons were carried out with the student’s *t* test. The data are expressed as the mean ± standard deviation. *p* values of <0.05 indicate statistical significance.

## Results

### Visualization of standardized surgical steps across two experienced surgeons enable rTLH workflow comparison

**Figure 2:**
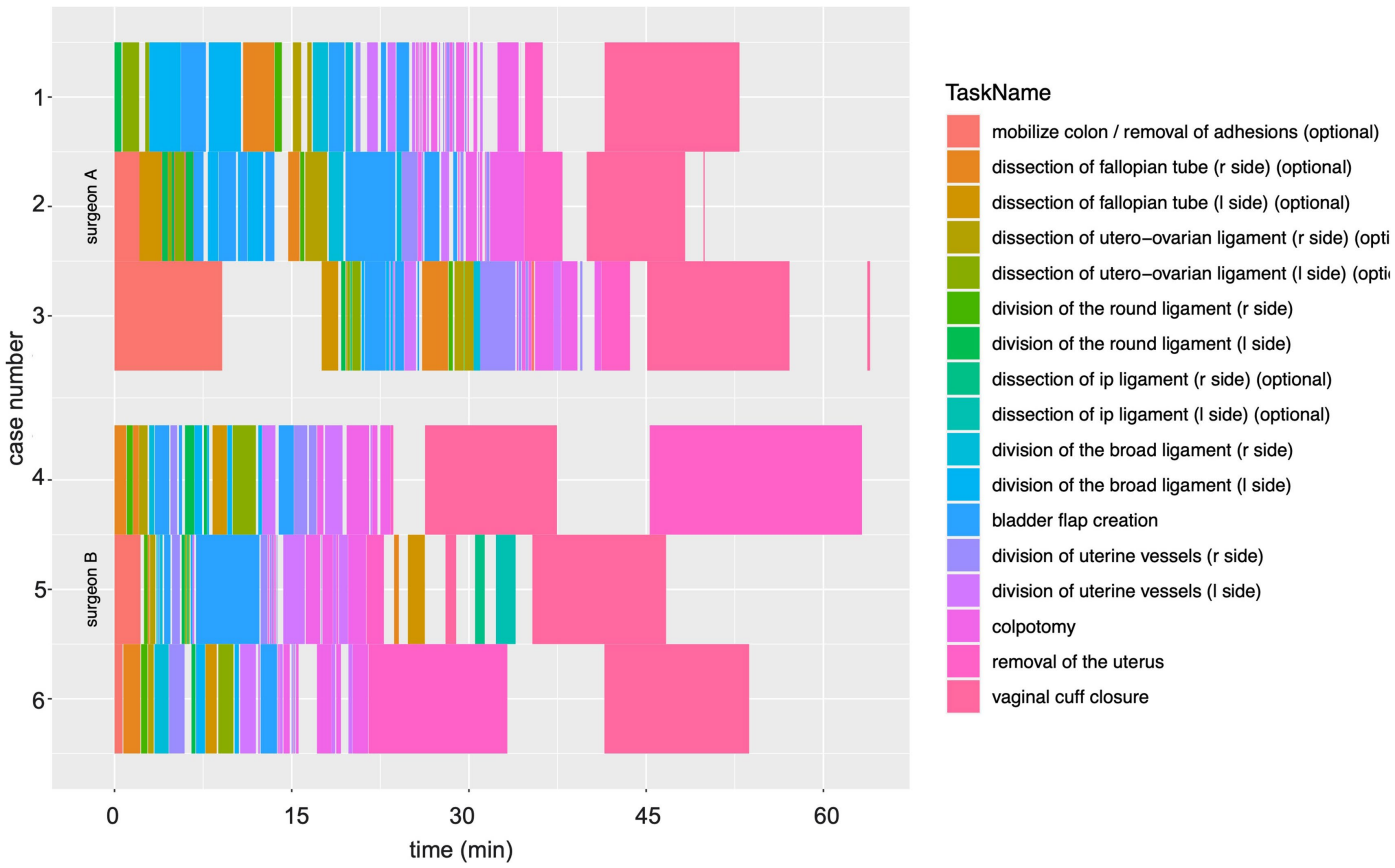
Visualization of surgical workflows across six unique rTLH cases enables objective workflow comparisons. Each color bar represents a surgical step with the length of the bar corresponding to its duration. Gaps between steps indicate idle time or surgical activity not aligned to the standardized hysterectomy steps. The existence of multiple bars of the same step name represent step switching by the surgeon or pause from the surgeon. All cases exhibited step switching and lapses between surgical steps, with each case exhibiting an individualized signature, enabling insights into surgical technique and workflow.

Individual steps across six rTLH cases from two experienced surgeons, who performed more than 30 TLHs using the da Vinci surgical system, were segmented, plotted, and compared (Figure 2). Case numbers 1–3 were performed by surgeon A, while cases 4–6 were performed by surgeon B. Patient characteristics are displayed in Table 2. By displaying each individual step over time, it is possible to observe the sequence of surgery exactly. Roughly similar sequence patterns were observed across the individual surgeries and across the two surgeons, with dissection of the fallopian tube followed by dissection of the round ligament, dissection of the broad ligament, bladder flap creation, division of the uterine vessels, colpotomy with removal of the uterus, and vaginal cuff closure using a barbed thread (Figure 1). An obvious difference observed was that surgeon A began with the hysterectomy on the left side, while surgeon B began on the right side. Despite the side differences on which surgery was stared, hereafter the surgical steps of all 6 surgeries follow the standard surgical steps described in the annotation card (Table 1—steps 5 to 11).

**Table 2:** Patient characteristics.

Pat. No.	1	2	3	4	5	6
Surgeon	A	A	A	B	B	B
Indication	Adenomyosis Fibroids	Fibroids	Fibroids	Fibroids	Fibroids	CIN III, Hypermenorrhea
Age (years)	47	44	45	45	58	49
BMI (kg/m^2^)	22.3	21.8	20.5	38.5	35.3	21.0
Uterus weight (gram)	240	368	202	375	104	130
Duration (min)	75	72	85	103	82	65
Special feature	Peritoneal Endometriosis	–	Adhesions, Ovarian cyst	Laparoscopic morcellation	Adnexectomy, Adhesions	Vaginal morcellation, Deep infiltration Endometriosis: Adhesions
Previous abdominal surgeries	Diagnostic laparoscopy	–	Cesarean section, Transversal laparotomy because of adhesions	Laparoscopic cholecystectomy	Cesarean section	–

Additionally, all cases display both blank time periods with no surgical steps indicated and surgical step switching. Blank periods occur when the change between surgical steps last longer than two seconds, e.g., due to change of instruments, need to obtain additional equipment such as retrieval bags or morcellators, or idle time. Gaps were observed in all six surgeries. Surgical step switching in all cases indicates that although a general workflow can be observed, the surgeon would perform a step for a certain duration, then return to that same step, displaying unique workflow signatures.

Individual differences across cases can be observed, as well. In surgery no. 3, extensive adhesiolysis was performed at the beginning of the procedure (Figure 2, broad orange bar). This may not be required in all cases, as variable presence of adhesions may eliminate the need for this step. Additionally, surgery no. 4 exhibits a longer duration for removal of the uterus, which follows the colpotomy. This case represents a patient with a large uterus myomatosus, which could not be retrieved through the vagina, so a laparoscopic morcellation was performed. This is similar to surgery no. 6, although the removal of the uterus is in reverse order to surgery no. 4. In this particular surgery, a vaginal morcellation was performed for large uterus myomatosus followed by laparoscopic suturing of the colpotomy. Together, this visualization uncovers both similarities and differences in individual hysterectomies and reflects unique components to aide in analysis of surgical workflow. Patient characteristics are displayed in Table 2.

### Step transition probabilities elucidate consolidated workflows of rTLH

**Figure 3:**
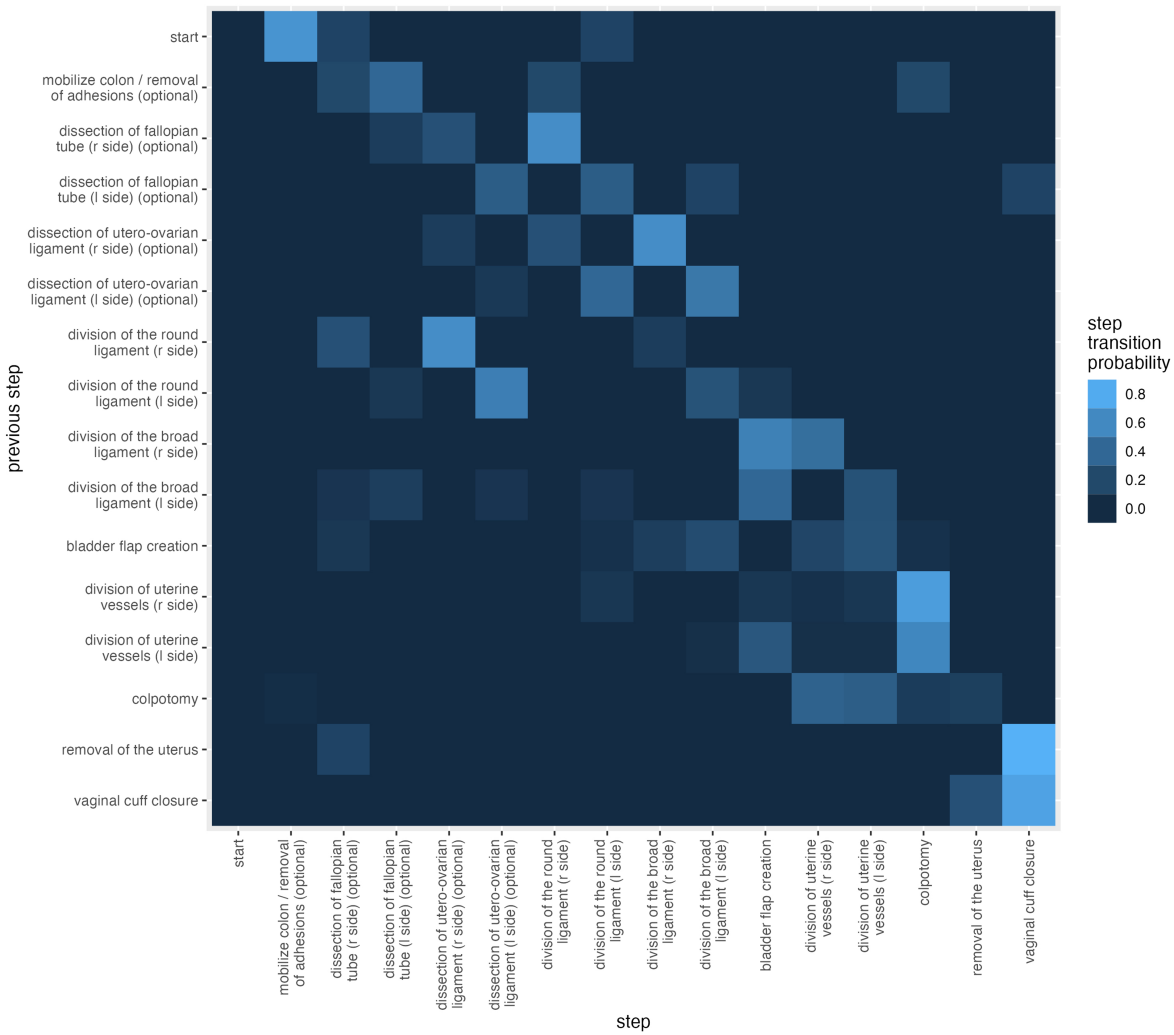
Step transition probability from six procedures from two surgeons combined. The ordering is right/left for paired steps.

Figure 3 shows the probability of the sequence of the surgical steps of all six rTLH surgeries. Lighter squares indicate a higher probability and black indicates that the transition never occurred. All surgeries start with the default “start” step and then the next step with highest probability is “mobilize colon/removal of adhesions (optional),” but “dissection of fallopian tube (r side) (optional)” and “division of the broad ligament (r side)” also occur with smaller probability. As most surgeries end with the vaginal cuff closure this square is the lightest in the last line. In one case the uterus was morcellated due to the size after the vaginal cuff closure, so there is also a square indicating the probability of the removal of the uterus which is darker than the one for vaginal cuff closure.

Surgical step order was determined from the calculated median fractional step order, but if a step had both a left and a right variant (e.g., Division of the Broad Ligament), then right was ordered before left. This fixed ordering provided consistency, as the right and left variants of a step had similar fractional step orders. Dissection of the infundibulopelvic ligament was excluded in this figure, as this step occurred only twice, once left and once right in case 5. A linear progression of the individual surgical steps from the upper left to the lower right corner of the diagram is apparent. Deviations from the direct diagonal indicate deviation from the standardized sequence of the individual surgical steps.

Note that the center diagonal, consisting of a surgical step followed by the same surgical step, is almost black with the exception of “vaginal cuff closure” indicating, that there was an alternation of steps. The parallel diagonal lighter lines beside the “black” line indicate a tendency of surgeons to keep working on the same side.

### OPI comparisons for colpotomy and vaginal cuff closure

**Figure 4:**
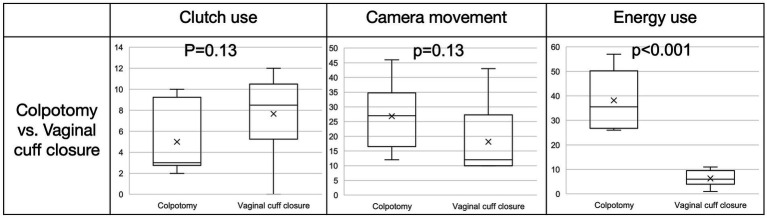
Whisker box plot. Comparison of event counts (clutch use, camera movement and energy use) between colpotomy and vaginal cuff closure for all six surgeries.

OPIs have been shown to provide insight into intraoperative surgeon behavior (13, 14). In addition to comparison of surgical step duration and workflow, data captured directly from the robotic data streams, such as events of energy use, clutching behavior, and camera movements were calculated into OPIs and observed. For detailed analysis of OPIs we choose the two most complex and standardized surgical steps of TLH: colpotomy and vaginal cuff closure. When comparing colpotomy to vaginal cuff closure for all six surgeries time (seconds) needed for the step (281.1 ± 83.7 vs. 677.5 ± 81.0) and the events of energy use (38.2 ± 11.3 vs. 6.3 ± 3.1) were significantly different (*p* < 0.001) (see Figure 4). For the events of camera movement and clutch use no differences were observed.

**Figure 5:**
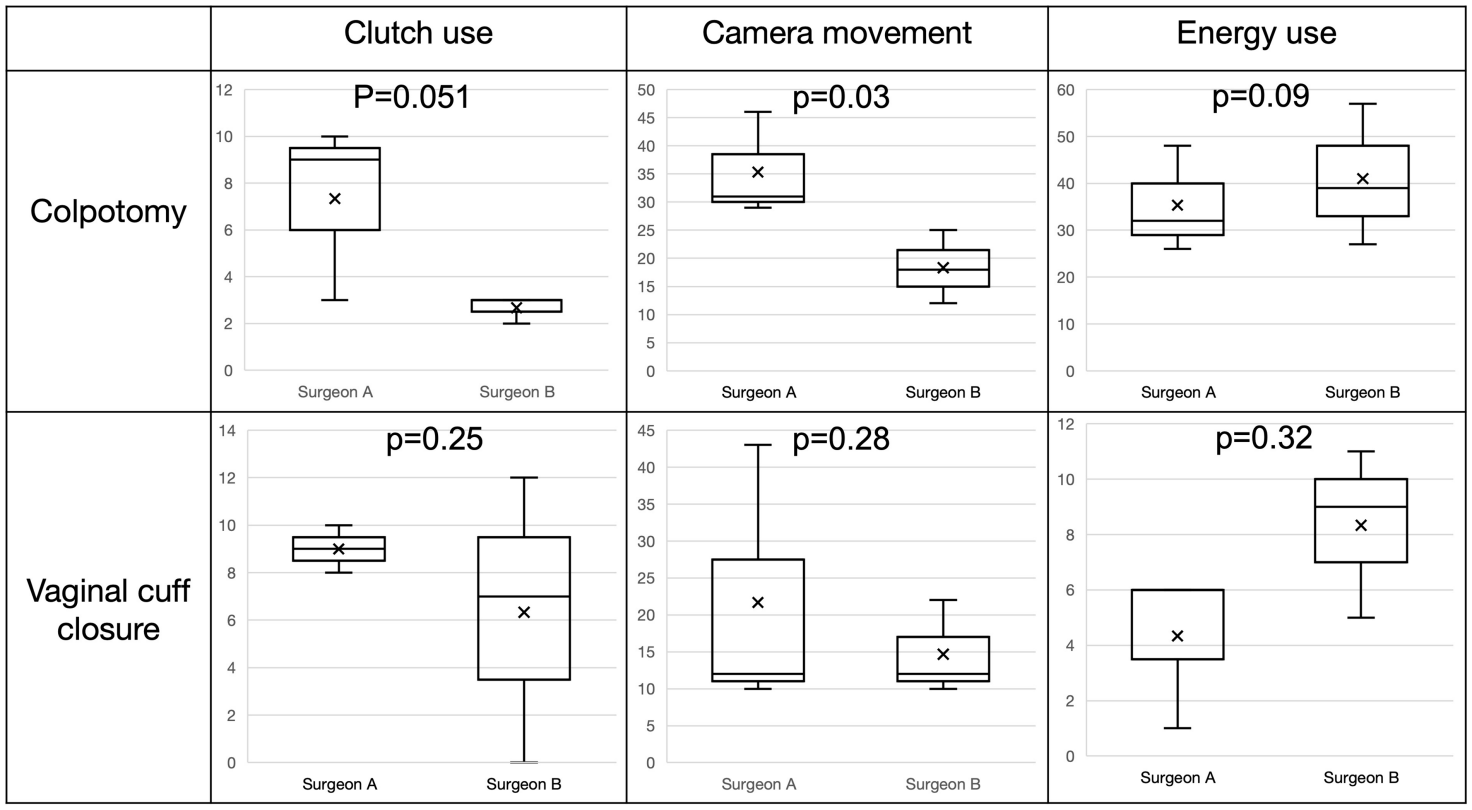
Event counts for clutch use, camera movements, and energy use can be compared across surgeons for specific steps. Box and whisker plots comparing event counts of clutching, camera movements, and energy use during colpotomy (top) and vaginal cuff closure (bottom) for Surgeon A and B during rTLH. “*x*” represents mean.

Importantly, OPI differences between the two surgeons were observed (see Figure 5). In comparable time of the respective steps, the event count for camera movement varies significantly between the two surgeons during the colpotomy step (surgeon A: 35.3 ± 7.6, surgeon B: 18.3 ± 5.3; *p* = 0.03) but not for vaginal cuff closure (surgeon A: 21.7 ± 15.1, surgeon B: 14.7 ± 5.3; *p* = 0.28). Clutching event count (addition of event clutch left, right and both) showed no significant differences between the two surgeons performing colpotomy and vaginal cuff closure (colpotomy: surgeon A: 7.3 ± 3.1, surgeon B: 2.7 ± 0.5; *p* = 0.051 and vaginal cuff closure: surgeon A: 9.0 ± 0.8, surgeon B: 6.3 ± 4.9; *p* = 0.25). Use of energy during colpotomy and vaginal cuff closer showed no significant difference between the two surgeons (colpotomy: surgeon A: 35.3 ± 9.3, surgeon B: 41.0 ± 12.3; *p* = 0.09 and vaginal cuff closure: surgeon A: 4.3 ± 2.4, surgeon B: 8.3 ± 2.5; *p* = 0.32), despite energy use was, as typical for this step, more frequently during colpotomy. Outliers in the frequency of current application indicate difficulty in hemostasis at the vaginal edges. Although the difference in clutching during colpotomy is not significant, there is a trend towards more actions for surgeon A, which provides preliminary feasibility for the need for future investigation.

## Discussion

This study provides the gynecology field a novel methodology to investigate OPIs in rTLH. We utilize this method to compare surgical workflows and surgeon behaviors across cases and surgeons. We show that annotation and visualization of independent surgical steps enables workflow comparisons across individual surgeries, as well as identification of OPIs differences and similarities in surgeon behavior. This could be a tool to monitor and adjust learning plans for gynecologic robotic surgery trainees. Together, this work lays the foundation for future gynecology studies using case segmentation and OPIs.

Hysterectomy is a highly standardized operation with fixed sequential surgical steps (15). As such, it was an ideal model to visualize surgical workflow changes for a small data set across two surgeons. In the step transition probability analysis (Figure 3), a clear workflow can be seen reliably from step to predicted step, supporting the high standardization of this procedure. We do observe a slight divergent dimming in the middle of the heat map, illuminating two distinct surgical approaches across surgeons; Surgeon A began hysterectomies on the left side and Surgeon B on the right side. Together, this work provides feasibility for unique surgical workflow signatures, which could be used for training, identification of complex cases, surgical techniques, and more. Metchik demonstrated that using a forward and backward entropy, similar to our model, behavioral patterns in the change between individual surgical steps can be shown in order to improve learning curves and workflows (16).

In this study, we analyzed the two most standard surgical steps in rTLH in detail: colpotomy and closure of the vaginal cuff. Since the circumference of the vagina must be viewed during the colpotomy, this surgical step exhibits high counts of camera movements. The use of energy is also high during this step as the vagina is opened by using monopolar energy. Despite both surgeons being experienced in rTLH, remarkably, clear OPI differences were observed. Thus, with the help of OPIs, not only can surgical workflow be elucidated, differences in surgical techniques and preferences can be identified and compared. In the future, it may be possible to distinguish between different surgeons and different surgeries based on the analysis of OPI signatures alone.

Our proposed methodology may enable tailored gynecology learning plans and opportunities to track learning progress. Although this study compared surgeries performed by experienced surgeons, it is likely that there will also be differences between experts and trainees, as has been shown in previous OPI studies in other specialties (10, 13). Other studies have shown the potential for similar metric data to track learning progression, such as Turner’s et al work in simulator studies (17) and Ma’s et al work in tissue models (18). These and other investigations of OPI utility have been increasing for the past 5 years, showing promise for surgical workflow analysis, training, skill, and correlation to patient outcomes (18, 19). This work is most prevalent in urology (13, 14, 18, 19), with limited published work emerging in the thoracic (9, 10) and general surgery specialties (8, 11, 20). However, no studies to date have utilized such technologies and methodologies in gynecology. As such, much work is needed in clinical gynecology cases to validate this potential.

International societies such as the European Society for Gynaecological Endoscopy (ESGE) have recognized the importance of developing structured training in robotics and have drawn up their own curriculum (Gynaecological Endoscopic Surgical Education and Assessment—GESEA programme) (21). In the future, analyses of segmented videos and OPIs could become part of such a structured learning concept in order to objectively quantify learning progress and possibly compare it with a large group of robotic surgeons.

The addition of visualization of surgical workflows, as shown in Figure 2, to the analysis of OPIs provide the additional advantage of identifying difficult situations during the surgery. This can be helpful in monitoring learning progress. Unusual situations can be reviewed retrospectively using video to identify deviations from the standard to fine tune training.

Although we present significant advantages to OPI evaluations, this study has a number of limitations. It must be noted that this work is a feasibility study on the use of a recorder for video and metric data for the use of the da Vinci Surgical System, which initially covers only six surgeries. Further studies are needed to support the data presented here and pave the way for routine use of OPI measurements. Additionally, this study does not utilize any trainees or surgeons that are in the initial stages of their learning curve, which will be critical for future studies. Although we evaluate surgeon behavior use as it pertains to camera manipulation, energy use, and clutching, we did not investigate any kinematic indicators of performance, which will be needed to elucidate surgeon behaviors in the future. Since a barbed thread was used for the vaginal flap closure, the knotting, which requires special fine motor skills, was not part of the study.

Together, the foundation laid in this work opens the door to countless and critical future investigations for truly objective characterizations and inquiry of intraoperative surgical behaviors, which can be used to train exceptional surgeons objectively and efficiently, leading to better patient outcomes.

## Conclusion

This pilot study presents a novel methodology to analyze and compare individual hysterectomy procedures across surgeons with truly objective measurements. Through collection of robotic data streams and standardized segmentation of hysterectomy cases, OPI measurements for specific rTLH surgery steps can be reliably calculated and compared to those of other surgeons. Utilization of this methodology provides opportunity for critical standardization to the gynecology field, which could be integrated into individualized training plans in the future. However, more data is needed to establish context surrounding these metrics as they pertain to gynecology.

## Data availability statement

The original contributions presented in the study are included in the article/supplementary material, further inquiries can be directed to the corresponding author.

## Ethics statement

The studies involving humans were approved by the Ethics Committee of Tübingen University Medical Faculty and University Hospital. The studies were conducted in accordance with the local legislation and institutional requirements. The participants provided their written informed consent to participate in this study.

## Author contributions

FN: Conceptualization, Data curation, Formal analysis, Project administration, Writing – original draft. SB: Conceptualization, Writing – review & editing. AB: Writing – review & editing. MS: Writing – original draft, Writing – review & editing. LP: Data curation, Formal analysis, Writing – review & editing. XL: Data curation, Formal analysis, Writing – review & editing. ME: Data curation, Formal analysis, Writing – review & editing. CW: Writing – review & editing. TD: Data curation, Formal analysis, Visualization, Writing – original draft. CR: Writing – review & editing. BK: Conceptualization, Writing – review & editing.
